# Conventional thyroidectomy: what is the impact of the scar on the lives of operated patients?

**DOI:** 10.20945/2359-3997000000379

**Published:** 2021-06-29

**Authors:** Gustavo Henrique Pereira Boog, Júlia Adriana Kasmirski, Flávio Carneiro Hojaij

**Affiliations:** 1 Universidade de São Paulo Faculdade de Medicina da São Paulo SP Brasil Faculdade de Medicina da Universidade de São Paulo (FMUSP), São Paulo, SP, Brasil.; 2 Faculdade de Medicina da Universidade de São Paulo Laboratório de Investigação Médica Departamento de Cirurgia São Paulo SP Brasil Departamento de Cirurgia, Laboratório de Investigação Médica (LIM 02), Faculdade de Medicina da Universidade de São Paulo (FMUSP), São Paulo, SP, Brasil.

**Keywords:** Thyroidectomy, surgery, scar, aesthetics, social impact

## Abstract

**Background:**

This study was aimed at investigating the aesthetic impact of scars on the lives of patients who undergo conventional thyroidectomy.

**Materials and Methods:**

This cross-sectional study was based on a retrospective analysis of 98 electronic medical records of patients who underwent conventional thyroidectomy performed by the same surgeon. The impact was determined through a qualitative question and categorized into three levels of dissatisfaction.

**Results:**

Among the 98 patients, 96 (97.95%) reported experiencing no functional or visual discomfort with their scars. The two unsatisfied individuals were women, and both classified their discomfort as moderate. Although the diseases that indicated surgery varied, papillary thyroid carcinoma predominated.

**Conclusion:**

The sample’s satisfaction level indicates that, in line with the current literature, the decision to opt for cosmetically appealing methods is not justified by aesthetic complaints about scars. The benefits of lower cost and fewer complications make conventional thyroidectomy an old but reliable option for afflictions of the thyroid gland that require surgery.

## INTRODUCTION

The anatomical description of the thyroid gland began in 1500 with Leonardo da Vinci. Nonetheless, until the 19th century, thyroidectomy was associated with high levels of complications such as dyspnea and difficulty breathing, hoarseness, hemorrhage, and infection. At the beginning of the 1850s, thanks to the parents of thyroidectomy, Theodor Billroth and Theodor Kocher, there was a remarkable methodological improvement in terms of antisepsis, vascular clamping, dissection techniques, and anesthesia (1). The method these pioneers created effectively reduced mortality from 40% to 2.4%. It consisted of a transverse incision starting at the anterior border of the sternocleidomastoid muscle and continuing up to its contralateral side (2). To minimize scar visibility, the incision is performed on the natural necklines. However, some problems persisted, such as the tetany caused by the loss of the parathyroids. These complications were systematically reduced by Anton Wolfer and Jan Radecki (3). Throughout the 20th century, new and better tools were created to guide and assist surgical results, such as histopathology and drugs intended to replace thyroid hormones (4).

The current trend consists of attempts to improve aesthetic aspects, decrease the length of hospital stays, and reduce possible complications. In this sense, minimally invasive thyroidectomy techniques were created, usually following the orientation of incisions smaller than 3 cm (5).

Among the described methods, minimally invasive video-assisted thyroidectomy (MIVAT) stood out. This technique, which consists of a 1.5-cm transverse incision 2 cm above the sternal manubrium, has limitations and is not indicated for patients with thyroid volume greater than 30 mL, nodules larger than 30 mm in diameter, or tumors larger than 20 mm (6). Another technique presented is endoscopy, which is performed by two periareolar incisions, followed by dissections and tissue detachments to reach the anterior neck, providing a view similar to that of open surgery. However, this method is associated with increased post-operative complications and discomfort due to the large area of displaced tissue (7).

The rise of robotic surgery introduced another technique involving a 5- to 6-cm incision in the posterior part of the pectoral muscle in the axillary region. Prohibited in the United States, this operation also has considerable limitations, such as the high costs, complications, and time of surgery (8). In the 2010s, a new method created by Anuwong constituted a relevant innovation. It is based on an incision made in the oral cavity, followed by dissection and detachment of the subplatysmal region and subsequent insufflation of the region with CO_2_, creating a space to operate from the upper border of the sternal manubrium to the oral vestibule (9).

The aesthetic appeal of these operative techniques is remarkable, especially concerning the absence of visible scars on the anterior neck region, but their limitations should not be ignored. For this reason, in this study, we investigated how much traditional thyroidectomy scars can socially and aesthetically impact the lives of patients who undergo this procedure.

## MATERIALS AND METHODS

In this cross-sectional observational study, we conducted a systematic and retrospective analysis of 98 electronic medical records of patients who underwent conventional thyroidectomy performed by the same surgeon at least six months before the interview.

To understand the impact of the scars resulting from these procedures on the patients’ quality of life, each person’s degree of satisfaction with their scar was explored in the medical records through the question “Does the scar bother you? If so, how much?” To categorize the complaints, the patients were asked to choose between “minimally” (no issues with the scar), “moderately” (self-conscious about the scar), or “considerably” (desire to change the scar).

The sample was composed of men and women, and the operative act was indicated due to the most diverse pathologies. There were no restrictions regarding age or any other selection criteria. Data regarding the patient’s characteristics were collected and included the following variables: sex, age, disease that motivated the surgery, subtype of thyroidectomy procedure (e.g., lobectomy, total thyroidectomy), surgery date, and last recorded consultation date.

The study was approved by the Research Ethics Committee of the Hospital das Clínicas da Faculdade de Medicina da Universidade de São Paulo (HC-FMUSP), São Paulo, Brazil (CAAE 40285320.1.0000.0068).

## RESULTS

**Figura 1:**
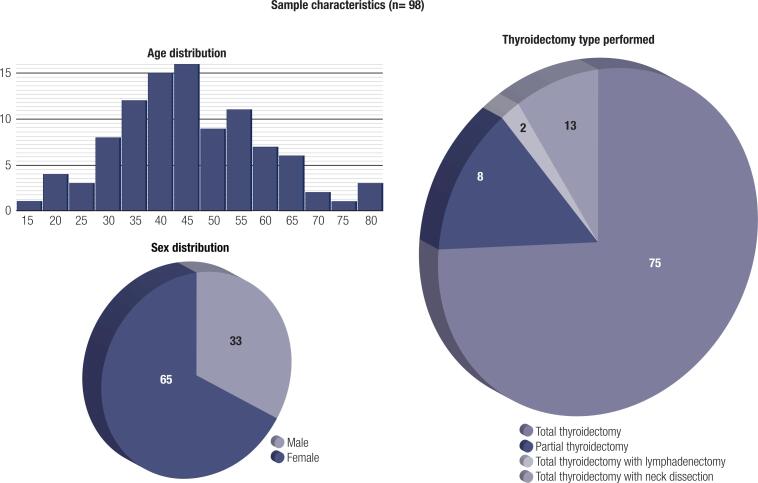
Sample characteristic graphs (age/sex distribution and thyroidectomy type performed).

Our sample included 98 Brazilian patients who underwent conventional thyroidectomy between 2003 and 2019. The mean age among the participants was 47.7 years (standard deviation = 13.7). The median age was 46 years (range 19-80). The group was composed of 33 (33.67%) men and 65 (66.32%) women, and 96 of the patients (97.95%) underwent medical monitoring in 2019, the year when the satisfaction data were collected. The conventional thyroidectomy models performed were as follows: 75 total thyroidectomies (76.5%), 8 partial thyroidectomies (8.2%), 2 total thyroidectomies with lymphadenectomy (2%), and 13 total thyroidectomies with neck dissection (13.3%). These sample characteristics results are shown in Figure 1. Although the diseases that indicated the surgery varied, papillary thyroid carcinoma predominated.

Among 98 patients, 96 (97.95%) reported experiencing no functional or visual discomfort with the scar. The patients reported to their surgeon. Both unsatisfied individuals were women submitted to total thyroidectomy, and both classified their discomfort as moderate.

**Figura 2:**
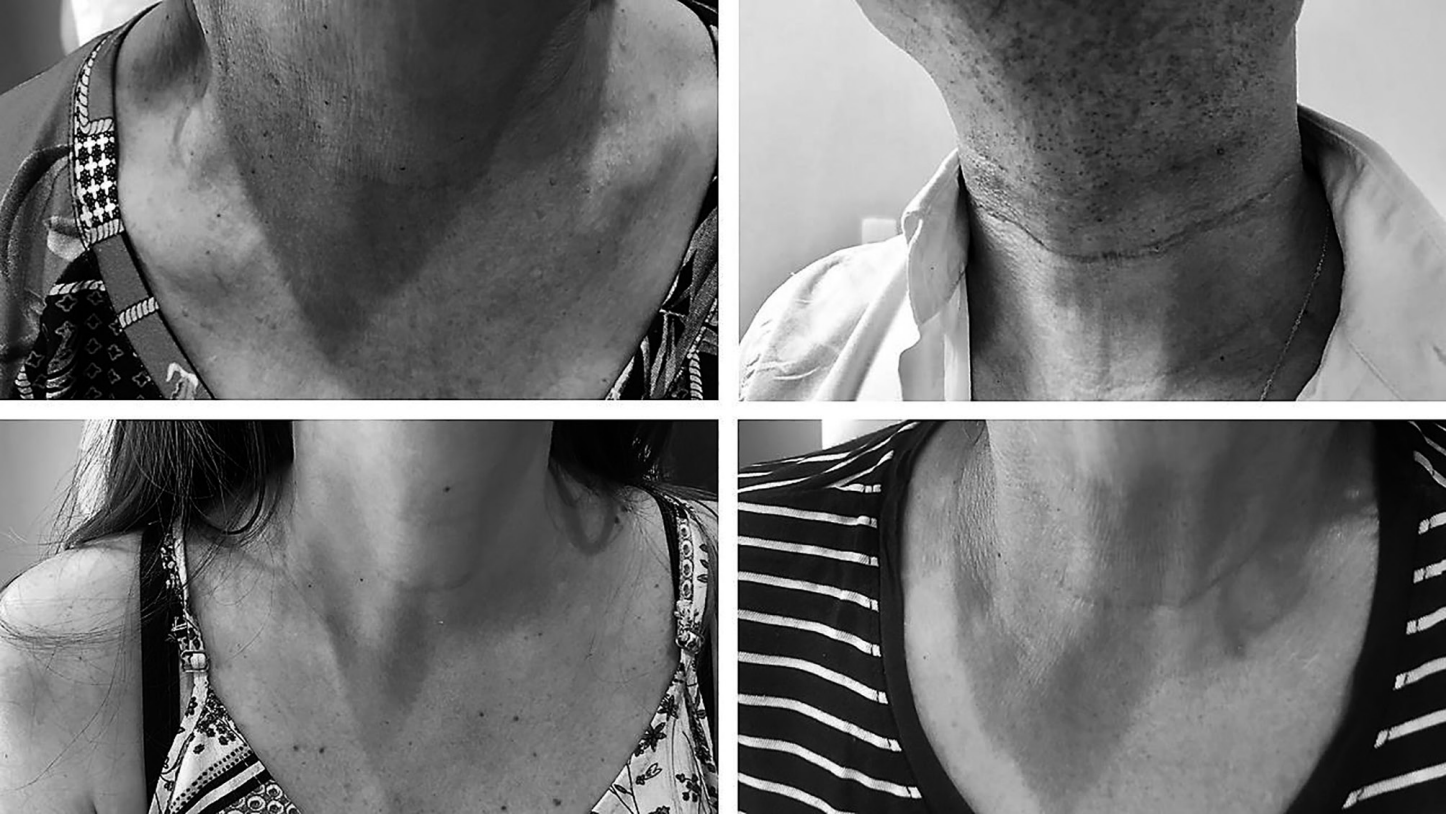
Four examples of patients satisfied with their conventional thyroidectomy scars.

All scars were inspected on physical examination. Twenty-one patients allowed the use of non-identifiable photographs to illustrate the results of their thyroidectomy scars. None of these individuals reported any discomfort. Four pictures were selected and included in Figure 2.

## DISCUSSION

The assessment of our sample’s general satisfaction and the current evidence (10) indicate that the decision to opt for minimally invasive methods, with their higher costs and longer operative times, is not justified by aesthetic complaints. In this regard, other factors gain importance, such as the experience of the surgeon, the length of the incision, and the post-operative care.

An interesting finding of our study was that the proportion of patients dissatisfied with the scars resulting from more invasive operations, such as associated neck dissection or lymphadenectomy, was not higher than that of the other members of the study.

The literature indicates that the social impact of the scar is minimal, as seen in approximately 90% of female patients, who did not feel the desire to hide the scar with accessories or request surgical revisions of the cicatrix (11). Indeed, the satisfaction rate in our investigation (97.85%) was similar to those reported in previous observational studies, such as 91.3% and 91.7% (11,12).

To optimize cosmetic outcomes, some general scar care guidance was provided to the patients. Silicon gel application on the incision region and specific massage techniques, such as circular manipulation, medial to lateral movements, and punctual compressions, are recommended for four months. In addition, the scar should be protected with sunscreen for at least one year.

There are some limitations to be considered. The sample was predominantly composed of women ages 40-60 affected by papillary carcinoma, and the low number of benign cases might influence the subjective perception of discomfort. In addition, we did not use any quantitative tool to assess cosmetic satisfaction, which might affect the study’s reproducibility.

Our study’s results indicate that conventional thyroidectomy remains the first choice of surgery for the most prevalent pathologies, carcinomas, and goiters of various categories, as well as any other thyroid diseases that cannot be resolved with pharmacological therapy. Even though the remote access techniques have an aesthetic appeal, the social and cosmetic implications of the conventional scar cannot justify these methods, because the cicatrix’s impact on the patient’s life is very low. The benefits of lower cost and fewer complications make conventional thyroidectomy an old but reliable option for afflictions of the thyroid gland that require surgical treatment.
